# Detection of Pathogenic Isoforms of *IKZF1* in Leukemic Cell Lines and Acute Lymphoblastic Leukemia Samples: Identification of a Novel Truncated IKZF1 Transcript in SUP-B15

**DOI:** 10.3390/cancers12113161

**Published:** 2020-10-28

**Authors:** Weiqiang Zhao, Ying Li, Chenjiao Yao, Guojuan Zhang, Kevin Y. Zhao, Wei Chen, Peng Ru, Xiaokang Pan, Huolin Tu, Daniel Jones

**Affiliations:** 1 The James Comprehensive Cancer Center and Solove Research Institute, The Ohio State University, Columbus, OH 43210, USA; guojuan.zhang@osumc.edu (G.Z.); zhao.1757@buckeyemail.osu.edu (K.Y.Z.); Wei.Chen@osumc.edu (W.C.); Peng.Ru@osumc.edu (P.R.); xiaokang.pan@osumc.edu (X.P.); Huolin.Tu@osumc.edu (H.T.); daniel.jones@osumc.edu (D.J.); 2 The Department of Pediatrics and Department of Hematology, Xiang-Ya Third Hospital, Changsha 410013, China; liying9771@csu.edu.cn (Y.L.); yaochenjiao@csu.edu.cn (C.Y.)

**Keywords:** ALL, IKZF1, isoforms, survival, Ik-6, Ik-8

## Abstract

**Simple Summary:**

Abnormal RNA splicing plays a fundamental role in leukemogenesis in acute lymphoblastic leukemia (ALL). Many cases of high-risk B-cell ALL cases, including BCR-ABL1+ and BCR-ABL1-like ALL, share a common molecular mechanism of aberrant fusion transcripts involving tyrosine kinase genes combined with dysregulation of the transcription factor and lymphocyte differentiation factor IKZF1. Dysfunction of *IKZF1* in ALL is caused by mutation and gene deletion but also alternative splicing resulting in exon skipping with production of aberrant IKZF1 proteins. We report here an assay to detect aberrantly spliced isoforms of *IKZF1* in ALL to assist in diagnosis, outcome prediction, and therapy selection in ALL and the identification of a novel altered IKZF1 product in a model ALL cell line.

**Abstract:**

Leukemia-associated alternative splicing of *IKZF1* can result in proteins with loss of one to four copies of its N-terminal zinc finger domains (N-ZnF). The best characterized pathogenic splice isoforms, Ik-6 and Ik-8, have been commonly found in BCR-ABL1+ acute lymphoblastic leukemia (ALL) and a subset of BCR-ABL1-like ALL. Infantile and childhood ALL that express these pathogenic IKZF1 isoforms have shown inferior clinical outcomes and can be resistant to tyrosine kinase inhibitors. Using ALL cell lines, we designed and validated a method to detect abnormal *IKZF1* transcripts. In the SUP-B15 leukemia cell line, we noted novel *IKZF1* transcripts that include both an Ik-6 splice and a transcript with a 14 base pair insertion at the C-terminus. There was also increased IKZF2 protein in SUP-B15 as compared to other ALL lines. Expression of Ik-6 could be suppressed by treatment with the pro-apoptotic type II histone deacetylase inhibitor givinostat. In 17 adult ALL samples, we noted the Ik-6 isoforms in 6 of 15 BCR-ABL1^−^, and 1 of 2 BCR-ABL1^+^ cases, with Ik-8 also expressed in one case. Cases with Ik-6 expression showed inferior survival as well as older age at presentation, lower expression of CD10 and more commonly a diploid karyotype.

## 1. Introduction

The Ikaros (*IKZF1*) gene codes for a C2H2-type zinc finger (ZnF) protein predominantly localized to pericentromeric heterochromatin in the nucleus and the histone deacetylase (HDAC)-containing the nucleosome remodeling and deacetylase (NuRD) complex and regulates many target genes essential for lymphocytic development and maturation [1,2,3]. It may also have a role in HDAC-independent transcriptional repression through the C-terminal binding protein (CtBP) co-repressor [4,5,6].

The *IKZF1* gene contains 8 exons comprising 6317 base pairs (bp), mapping to chromosome 7p12.2 [2]. Alternative splicing of *IKZF1* is a common post-transcriptional modification that can result in differential protein activity and function. At least 15 different *IKZF1* alternate splice products have been characterized that result in proteins that lack some of the N-terminal C2H2-type ZnFs with or without C-terminal alterations [2,7,8]. The wild-type IKZF1 protein has four N-terminal ZnF domains which mediate DNA binding and two C-terminal C2H2 ZnF motifs mediating dimerization [7]. The most well-characterized *IKZF1* alternate splice products are those that lack 3 (Ik-8) or 4 (Ik-6) N-terminal ZnFs. The altered proteins produced have been shown to function as dominant/negative inhibitors of the intact protein [3].

The pathogenesis of many cases of precursor B-acute lymphoblastic leukemia (B-ALL) can be linked to expression of Ik-6 or Ik-8. Using RT-PCR, Nakase et al. found overexpression of the dominant-negative Ik-6 isoform in 14 of 41 adult B-ALL but no other dominant-negative IKZF1 isoforms [9]. Ik-6 or Ik-8 have also been shown to be particularly common in BCR-ABL1-like ALL, a newly-recognized entity in the revised 2016 WHO hematopoietic/lymphocytic neoplasm classification [10]. In both adult and children BCR-ABL1+ ALL, cases expressing Ik-6 and Ik-8 *IKZF1* isoforms have been associated with a poor outcome [11,12,13]. The presence of Ik-6 and other pathogenic isoforms in adult B-ALL with other karyotypic findings is less common. In this study, we establish a rapid and reliable molecular assay to screen for Ik-6 and Ik-8 as well as for C-terminal ZnF size alterations and provide data on expression patterns in leukemia cell lines and adult ALL patient samples.

## 2. Results

### 2.1. Characterization of the Splicing Isoforms of IKZF1 in Leukemic Cell Lines

**Figure 1:**
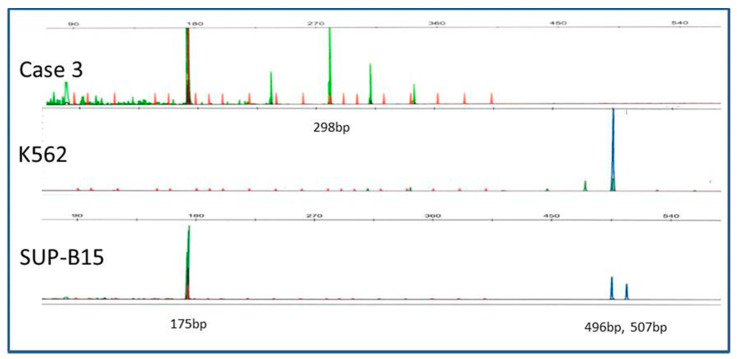
Detection of Ik-6, Ik-8, and other transcripts by size-fragment analysis. Multiplex RT-PCR with hexachlorofluorescein (HEX)-labeled (Ik-6/8 product) and 6-carboxyfluorescein (FAM)-labeled (IKT product) primers was performed using capillary electrophoresis. The samples included K562, SUP-B15 and an ALL sample (Case 3 or 3565. Table 1). Ik-6 and Ik-8 PCR products were detected at 175 bp and 298 bp, respectively. Other peaks in the ALL sample likely represent non-specific peaks or lower-level RNA isoform species but were not further characterized. The normal C-terminal IKT product was detected at 496 bp in K-562, with an additional abnormal 507 bp detected in SUP-B15, which was confirmed by DNA sequencing to represent a 14-base pair insertion (see text).

**Figure 2:**
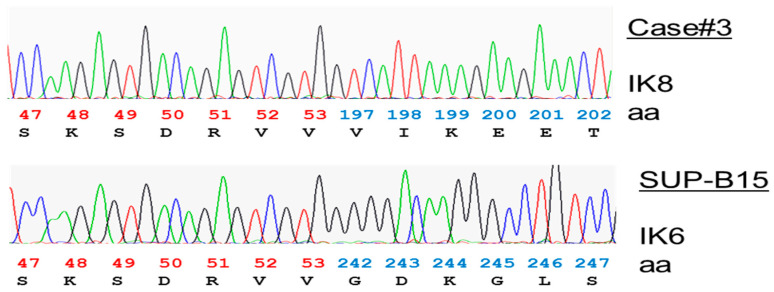
Sequencing confirmation and amino acid translation of the Ik-8 and Ik-6 PCR products. Sanger sequencing was performed on cloned Ik-8 and Ik-6 isoforms in a clinical sample (case 3565, Table 1) and lymphoblastic cell line (SUP-B15), respectively. The colored numbers below the sequencing traces were predicted codons and corresponding amino acids of IKZF1 (NC_000007.14).

To validate a rapid IKZF1 isoform detection method, RT-PCR was first performed on leukemia cell lines. With a hexachlorofluorescein (HEX)-labeled primer set that detects Ik-6/8 splice products, the BCR-ABL1+ SUP-B15 line showed a 175 base pair (bp) peak indicative of Ik-6 (Figure 1, lower panel), with no Ik-6 or Ik-8 products in other lines assessed (see Materials and Methods). Clinically defined ALL samples were then screened and one sample (3565, line 3 in Table 1) contained both Ik-6 and a 298 base pair indicative of Ik-8 (upper panel, Figure 1). To confirm the identity of these products, the corresponding bands were excised from a gel, cloned and sequenced. The sequencing results confirmed that the 175 bp-peak band in SUP-B15 was Ik-6 (reference sequence NM_001291840.1) with the expected junction (p.V53 of exon 3 fused with p.G242 of exon 7 and p.D243 exon 8) (Figure 2, lower panel). The 298 bp product in case 3565 showed the expected Ik-8 junction site (NM_001291843.1) with codon 53 in exon 3 (c.166-168/GAG) linked to codon 197 in exon 7 (c.724-726/GTC) (Figure 2, upper panel).

To screen for C-terminal IKZF1 insertion-deletion mutations, the cell lines and ALL samples were also screened with a 6-carboxyfluorescein (FAM)-labeled primer set producing a 496 bp wild-type product. The BCR-ABL1 + K562 showed only a single 496 bp peak but case 3565 lacked C-terminal peak, indicating C-terminal deletion (Figure 1, upper and middle panels). In addition to the 496 bp peak, an abnormal peak of 507 bp was detected in SUP-B15 (Figure 1, bottom panel). By DNA sequencing, this product had a 14 bp insertion (c.1334-1335 Ins(GGAACAATGGCCCC)), which is predicted to produce a truncated IKZF1 protein (p.N443fs*47, p.Thr215-p.Pro260*).

### 2.2. Identification and Characterization of Isoforms of IKZF1 Proteins in Leukemia Cell Lines

**Figure 3:**
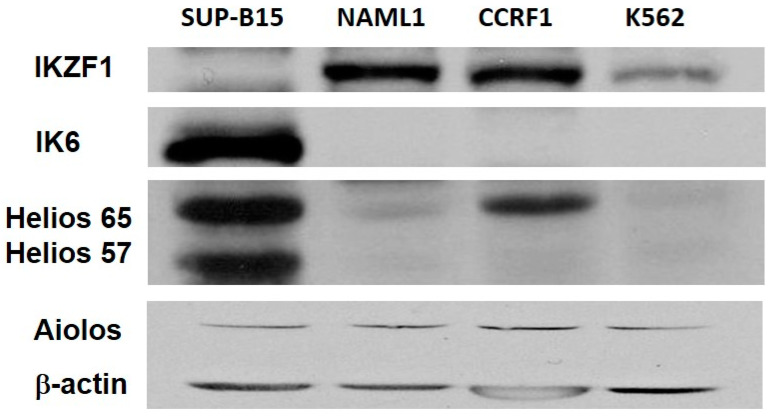
Detection of IKZF1 and IKZF family proteins in leukemic cell lines. Western blot analysis of four leukemia cell lines for IKZF1 full protein (top), Ik-6 (2nd from top), both with an anti-IKZF1 antibody, as well as Helios (anti-IKZF2 antibody), Aiolos (anti-IKZF3 antibody), and β-actin. The expected sizes of the proteins are IKZF1: 57.5 kDa, Ik-6: 38.2 kDa, Helios/IKZF2: 65 and 57 kDa, Aiolos/IKZF3: 70 kDa, and β-actin: 42 kDa (Figure S1).

Given the abnormal results seen by RT-PCR, the IKZF proteins produced in leukemic cell lines were characterized by Western blot analysis. The expected wild-type IKZF1 protein (57.5 kDa based on NP_001278769.1) was present in the NALM1, CCRF-SB, and K562 lines (Figure 3, Lane 2–4). In contrast, the wild-type IKZF1 protein was not detectable in SUP-B15, but a 38.2 kDa product was present, corresponding to the predicted size of an Ik-6 protein (Figure 3, Lane 1). Over-expression of IKZF2/Helios was observed in SUP-B15 as compared to three other leukemic cell lines whereas the expression levels of IKZF3/Aiolos were the same among all of 4 cell lines (Figure 3).

### 2.3. Localization of Ik-6 IKZF1 Proteins in SUP-B15

**Figure 4:**
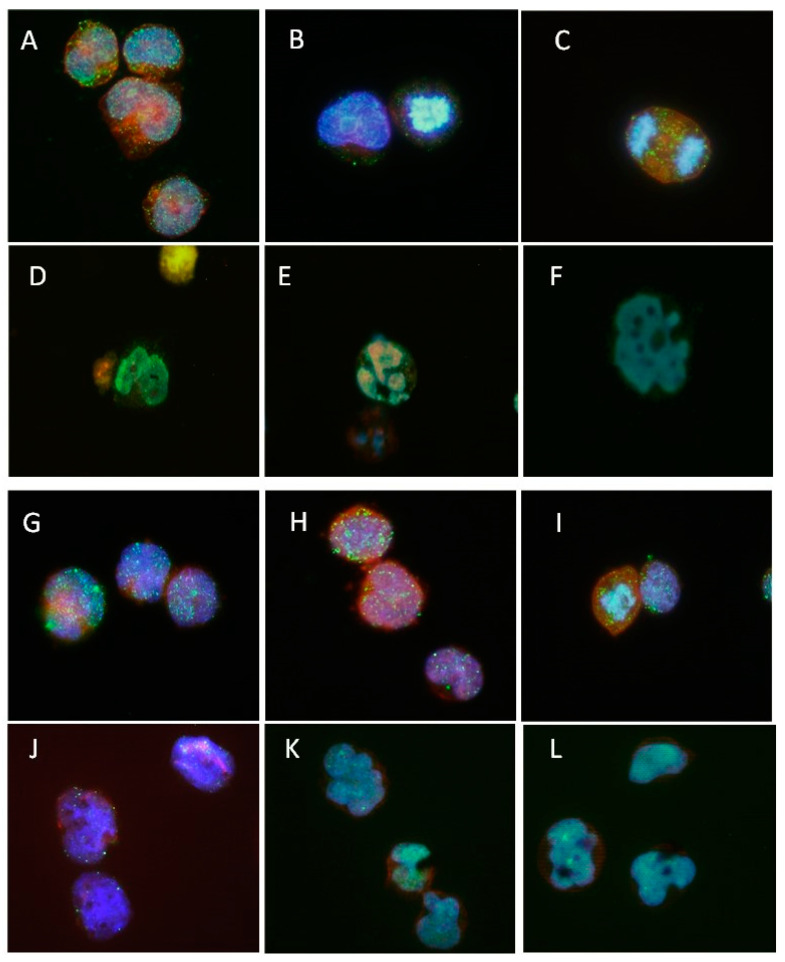
Immunofluorescence for IKZF1 in cultured SUP-B15 and NALM1. Untreated (**A**–**C**) and givinostat-treated (**D**–**F**) SUP-B15, and untreated (**G**–**I**) and givinostat-treated (**J**–**L**) NALM1 were subjected to immunofluorescence microscopy with representative cells shown for each group. The cytoskeleton (F-actin) was highlighted with Alexa 568 phalloidin (red), nuclei with DAPI (blue), and IKZF1 proteins with FITC-conjugated anti-rabbit antibodies (green). Image magnification (100× oil immersed fluorescence microscopy).

The altered Ik-6 is predicted to be defective in DNA binding due to its loss of all four N-ZnFs, with the additional C-terminal truncated protein expected to be deficient in multimerization. To assess these predictions, we performed immunofluorescence (IF) on SUP-B15 and NALM1 (Figure 4). In non-dividing cells, SUP-B15 and NALM1 cells show a similar distribution of IKZF1 protein in a speckled pattern in both cytoplasm and nucleus (Figure 4A,G,H). During mitosis, most of the Ik-6 protein in SUP-B15 was colocalized with the mitotic spindle (Figure 4C) but other protein remained dispersed through the cytoplasm, possibly reflecting the C-terminal truncated IKZF1.

We examined the effects on localization of IKZF1 in SUP-B15 (Figure 4D–F) and NALM1 (Figure 4J–L) cells following treatment with 0.5 μM givinostat for 24 h. Along with loss of IKZF1 and/or Ik-6 proteins in the leukemia cells, the nuclei underwent nuclear punctations as well as fragmentation reflecting apoptosis.

### 2.4. Correlation of IKZF1 Isoforms with Clinicopathologic Features in ALL

Following validation of the rapid assay for detection of Ik-6, Ik-8 and C-terminal indels, 17 cases of adult ALL were investigated (Table 1). These included 12 males and 5 females with a median age of 43 (from 20 to 77) years old. Immunophenotypically, 15 were classified as precursor B-cell and 2 with precursor T-cell ALL. The average blast percentage in the tested specimens was 64.1 ± 30.1 (95% CI: 49.1–79.1%). The B-ALL blasts all expressed cytoplasmic (c) cCD79a, CD34, TDT and negative for cCD3 and myeloperoxidase (cMPO). For T-ALL, they expressed cCD3, CD34, TdT, and were negative for cMPO. The t(9;22)(q34;q11) were detected in 2 B-ALL patients (2/17) and further confirmed to have BCR-ABL1 p190 transcripts.

Seven of the ALL cases (41.2%) showed Ik-6 and/or Ik-8 isoforms indicating that one or both IKZF1 alleles were abnormally spliced. Two of the cases (17.6%) also showed loss of the 496 bp IKT product supporting intragenic alterations of exon 8 of IKZF1.

Comparing presenting features with IKZF1 isoform type (Table 2), the Ik-6+ patients were significantly older (50 vs. 34, *p* = 0.021) and showed less CD10 expression (in B-ALL cases) than the cases without Ik-6 (32% vs. 61.4%, *p* = 0.041). The Ik-6+ ALL showed trends towards lower blast count (50% vs. 74%, *p* = 0.055), higher TdT (77% vs. 58.7%, *p* = 0.09), and less CD34 (59% vs. 80.1%, *p* = 0.161). Ik-6+ ALL more frequently had normal ploidy by cytogenetic analysis (14.2% vs. 72.7%, *p* = 0.005). The median survival time (months) of Ik-6+ patients was significantly shorter (17.3 months vs. 61.7 months, *p* = 0.022).

## 3. Discussion

In this study, using a rapid, inexpensive fragment analysis assay, we characterized IKZF1 alterations in leukemic cell lines and primary ALL samples.

We detected Ik-6 in 6 of 15 (40%) adult Ph-negative ALL samples and in 1 of 2 Ph-positive ALL patients. Even with this small sample, we were able to identify a number of statistically significant clinicopathologic features associated with Ik-6+ ALL, including older age at presentation, lower CD10 expression and normal ploidy by karyotyping. Most significantly, Ik-6+ ALL patients appeared to have inferior outcomes based on median survival time, paralleling what was seen in previous studies [11,12,13]. This consistent finding across multiple studies supports the conclusion that the altered function of IKZF1 produced by the Ik-6 splice can confer resistance to current ALL therapies.

To further studies on the function of Ik-6, we characterized the *IKZF1* transcript pattern in lymphoblastic leukemia cell lines and identified SUP-B15 as an Ik-6 bearing line that also shows an IKZF1 transcript with a C-terminal 14 bp frameshift insertion in exon 8 and produces only a truncated IKZF1 protein with no wild-type IKZF1 protein. Based on the presence of an intact C-terminal PCR product in SUP-B15, it is likely that the Ik-6 splice and the C-terminal defects are present on different alleles leading to the complete loss of wild-type protein function. Comparing the localization of wild-type IKZF1 (in NALM1) and Ik-6 or C-terminal-deleted proteins (in SUP-B15) by immunofluorescence microscopy, we noted the pattern in non-dividing cells was similar. In dividing cells, the majority of IKZF1 protein localized to the mitotic spindle indicating the general localization of the altered protein(s) was intact. SUP-B15 thus represents an ideal line to further study the functional differences between Ik-6-bearing and wild-type IKZF1 protein.

Previous studies have provided structural evidence for the implications of the Ik-6 splice on IKZF1 protein function but the effects of C-terminal alterations have been less studied. The two C2H2-like structures present in the end of the C-terminus have been shown to be critical for IKZF1 homo- or hetero-dimerization. In normal lymphocytes, IKZF1 and IKZF2 proteins are assembled into higher molecular weight multimers through C-terminal domain binding, and associate predominantly with subunits of NuRD, an ATP-dependent nucleosome-remodeling complex [14,15,16]. In addition, the intact C-terminal C2H2-domain plays a role in the dominant-negative effect with isoforms lacking DNA-binding domains, or pathogenic isoforms of IKZF1. Interestingly, in addition to altered IKZF1 transcripts (and no wild-type IKZF1 protein), SUP-B15 also showed strong upregulation of IKZF2/Helios. Experiments are underway to assess whether this represents a direct autoregulatory transcriptional effect of the altered IKZF1 proteins, as has been seen in some models [17].

Our previous studies have shown that brief treatment of SUP-B15 (and other acute leukemia cell lines) with givinostat results in significant cell growth arrest, activation of the caspase cascade and eventual apoptosis induction [18]. Our initial experiments have shown that givinostat also suppresses the expression of wild-type IKZF1 and the altered IKZF1 protein(s) in these two cell lines along with morphological changes of apoptosis. Further investigation on the connections between the action of givinostat and IKZF1 dynamics in SUP-B15 may help establish a rationale for givinostat therapy in Ik-6-bearing ALL.

## 4. Materials and Methods

### 4.1. Summary of Cell Lines and Ancillary Studies

The Buck Institutional Review Board (IRB) approved this study. Four acute leukemia (AL) cell lines, AML K562 (ATCC, CCL-243), B-ALL SUP-B15 (ATCC, CRL-1929), NAML1 (ATCC, CRL-1567), and CCRF-SB (ATCC, CCL-120), were obtained from American Type Culture Collection and maintained at PML as described previously [18]. Leukemic cells were also treated with 0.5 μM givinostat (Selleck Chemicals, Houston, TX, USA) for 24 h.

For ALL samples, morphologic features were assessed by Wright-Giemsa stained bone marrow aspirate and peripheral blood smears. All specimens were analyzed by flow cytometry using a standard ALL panel, conventional G-banded karyotyping and fluorescence in situ hybridization (FISH) for probes for BCR-ABL1 and MLL/11q23. Subtyping of BCR-ABL1 transcripts was performed by quantitative real-time PCR (RT-qPCR) on RNA extracted from leukemia cells as described previously [18,19]. The p210 BCR-ABL1 transcript was detected in K562 and NAML1 (B-ALL), and the p190 BCR-ABL1 transcript in SUP-B15 (B-ALL). CCRF-SB was negative for BCR-ABL1.

### 4.2. RNA Extraction, RT-PCR, and cDNA Sequencing

For IKZF1 studies, RNA was extracted from frozen cell pellets provided by the Leukemia Bank of the James Comprehensive Cancer Center of The Ohio State University. Reverse-transcription polymerase chain reaction (RT-PCR) was performed using two primer sets. Primers for Ik-6 or Ik-8 isoforms flanking exons 2/3 and exon 7 (NM_006060.5, NC_000007.14) were: F1, 5′-TACTCCAGATGAGGGCGATG-3′; and R1, 5′-CATCACGTGGGACTTCATCA-3′, which was predicted to produce either a 175 or 298 bp product for Ik-6 and Ik-8, respectively. The C-terminal portion of IKZF1 was using the IKT primer set with forward (F2) 5′-GAGCGCGAGGCGTCCCCGAG-3′ and reverse (R2) primers 5′-AGCAGCATAGACTGGACTGGA-3. For cloning, 5 μL of PCR products were run on a 2% agarose gel and stained with ethidium bromide, and the products representing Ik-6, Ik-8, and IKZF1 were excised from the gel, purified (QIAquick PCR purification kit; Qiagen, Santa Clarita, CA, USA) and cloned into PCR II (TOPO TA Cloning kit, Invitrogen, San Diego, CA, USA) as described previously [19]. Three colonies were selected from each band, DNA purified (QIAamp DNA Mini kit, Qiagen, Chatsworth, CA, USA) and sequenced using M13 forward and reverse primers on a 3130xl Genetic Analyzer. The sequences were aligned to IKZF1 cDNA sequence using NCBI-Blast.

Multiplex PCR reactions for Ik-6/8 and IKT were performed using primers modified with 5′-primer labeling with hexachlorofluorescein (HEX) or 6-carboxyfluorescein (FAM), respectively. The amplicons were subjected to capillary electrophoresis on 3130xl DNA analyzer (Applied Biosystems, Foster City, CA, USA).

### 4.3. Characterization of Ikaros Isoform (Ik-6) by Western Blot and Immunofluorescence Analysis (IF) on Cultured Leukemic Cell Lines

The Western blot was performed as described previously [18]. For IF, cultured SUP-B15 and NALM1 leukemia cells were harvested at 24 h treated or untreated with 0.5 μM givinostat. The cells were washed twice with PBS and re-suspended to approximately 500–1000 cells in 100 to 250 μL, and spread onto a polylysine-treated cover slide. The cells were fixed and permeabilized in 4% formaldehyde/0.1% Triton-X 100 in 1 × PBS with 5% (*v/v*) Alexa568 phalloidin (Thermo Fisher Scientific, Waltham, MA, USA) for 5 min at RT. The fixed slides were washed with 1 × PBS/0.05% Tween-20 for 5 min × 3, and kept at 4 °C before stain. The slides were rehydrated with borohydride solution (1 mg/mL NaBH4 prepared freshly in 1 × PBS) for 10 min at RT, and blocked with 5% fetal bovine serum/0.2% Tween 20/PBS for 30 min, at 37 °C. The slides were incubated with anti-IKZF1 (1:500), Novus, rabbit polyclonal diluted in BSA/PBS (1:50, (2%)) for 30 min at 37 °C. The slides were washed with 1 × PBS/0.05% Tween 20 for 5 min and repeated three times to remove unbound antibodies. The slides were further incubated with FITC coupled anti-rabbit Ig (1:100) and 5% (*v/v*) Alexa568 phalloidin in BSA/PBS (1:50) for 30 min at 37 °C and washed three times with 1 × PBS/0.05% Tween-20. Nuclear staining was performed using 10 ng/mL DAPI (H-1500, Vector Laboratories, Burlingame, CA, USA) solution for 5 min, and rinsed once with 1 × PBS/0.05 Tween 20. The slide was desalted by quick dipping stained slide in dH_2_O and covered with cover slide in 1 × PBS/glycerol (1:1) and sealed with nail polish. Images were captured using fluorescence microscope (Olympus BX51, Center Valley, PA, USA) equipped with three filters, with 30-150132 for orange signals, U-NSP101 for green, and 30-158232 for combined/triple (red, green and DAPI) signals.

Statistic studies: Patient categorical data are expressed as mean or median ± SD. Student *t*-test/Kruskal-Wallis tests were performed between different groups and differences are considered as statistically different when *p*-value < 0.05.

## 5. Conclusions

In summary, we characterized an Ik-6, Ik-8, and novel C-terminal frameshift insertion transcript in the SUP-B15 leukemic cell line that produces an Ik-6, and predicted novel truncated IKZF1 product. We also characterize the clinicopathologic features that distinguish Ik-6/Ik-8+ ALL from those leukemic samples that lack these isoforms in an adult cohort.

## Figures and Tables

**Table 1:** Screening of IKZF1 isoforms expressed in ALL samples.

Individual ID	Gender	Age	Diagnosis	Blasts	BCR-ABL1 ^#^	Isoform *	C-Terminus (496 bp)
1862	M	21	B-ALL	85%	absent	wt.	loss
2148	M	38	B-ALL	31%	absent	Ik-6	present
3565	M	77	B-ALL	20%	absent	Ik-6, Ik-8	loss
4139	M	50	B-ALL	45%	absent	Ik-6	present
5735	M	59	B-ALL	24%	detected	Ik-6	present
6026	F	48	B-ALL	88%	absent	wt	present
7076	M	34	B-ALL	46%	absent	wt	present
8888	F	53	B-ALL	95%	detected	wt	present
9862	M	21	B-ALL	85%	absent	wt	present
10585	F	43	B-ALL	73%	absent	Ik-6	present
11721	M	20	B-ALL	96%	absent	wt	present
12083	F	52	B-ALL	63%	absent	wt	present
14355	M	27	B-ALL	50%	absent	wt	present
15086	M	48	T-ALL	97%	absent	wt	present
16380	F	56	B-ALL	94%	absent	Ik-6	present
17433	M	36	B-ALL	63%	absent	Ik-6	present
18972	M	34	T-ALL	50%	absent	wt	present

^#^ p190 (e1a2) detected by qRT-PCR. * wt: wild-type (496 bp fragment only); Ik-6: 175 bp product; Ik-8: 298 bp product.

**Table 2:** Clinicopathologic correlations between Ik-6 and non-Ik-6-expressing ALL.

Features	Ik-6 Not Detected	Ik-6 Detected	*p*-Value
Median age at presentation (range)	34 (20–53)	50 (36–77)	0.021
% Blasts (mean ± SD)	74% (31)	50% (28)	0.055
CD10% (mean ± SD)	67% (45)	32% (31)	0.041
CD34% (mean ± SD)	78% (28)	59% (44)	0.161
TdT% (mean ± SD)	57% (34)	65% (17)	0.086
Hyperploidy (%)	80% (8/10)	29% (2/7)	0.022
Median survival in months, range	61.7 (11.8–89.4)	17.3 (0.4–83.3)	0.022

## Supplementary Materials

The following are available online at https://www.mdpi.com/2072-6694/12/11/3161/s1, Figure S1: Uncropped Western Blotting Figures.
