# Supplementary material for: Detection of Pathogenic Isoforms of IKZF1 in Leukemic Cell Lines and Acute Lymphoblastic Leukemia Samples: Identification of a Novel Truncated IKZF1 Transcript in SUP-B15
Source: Cancers (Basel). 2020 Oct 28;12(11):3161. doi: 10.3390/cancers12113161 (PMC7693987; doi:10.3390/cancers12113161)
Supplement: Supplementary file 1 [file cancers-12-03161-s001.pdf]

cancers

MDPI

Supplementary Materials

Detection of Pathogenic Isoforms of *IKZF1* in Leukemic Cell Lines and Acute Lymphoblastic Leukemia Samples: Identification of a Novel Truncated IKZF1 Transcript in SUP-B15

Weiqiang Zhao, Ying Li, Chenjiao Yao, Guojuan Zhang, Kevin Zhao, Wei Chen, Peng Ru, Xiaokang Pan, Huolin Tu and Dan Jones

A

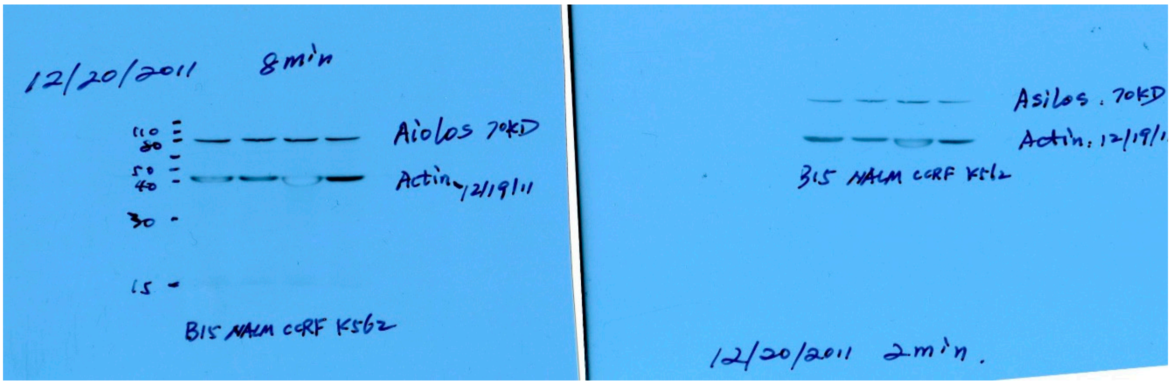

B

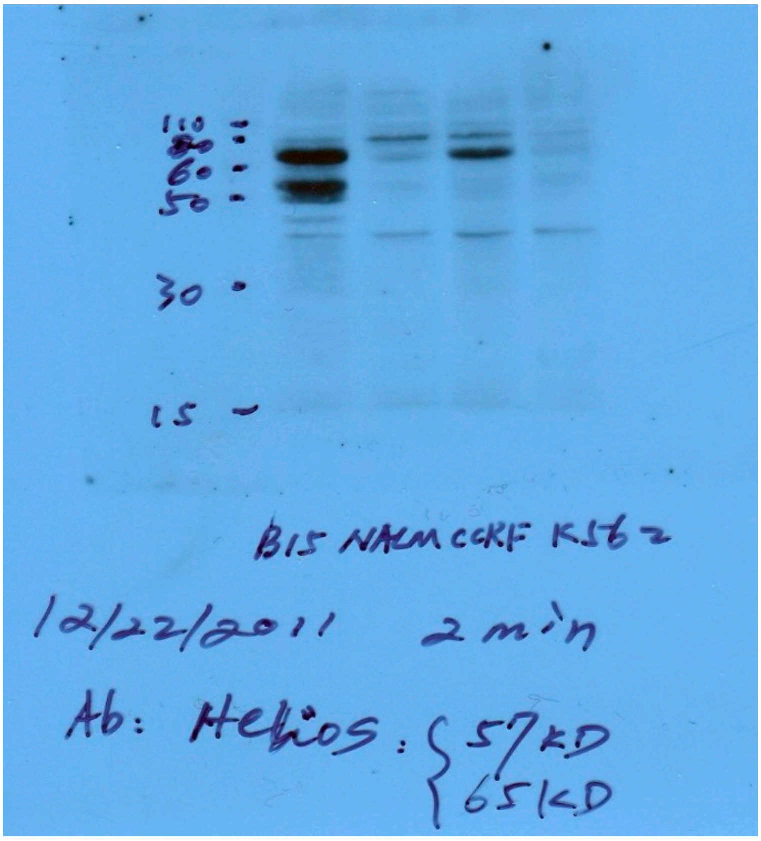

C

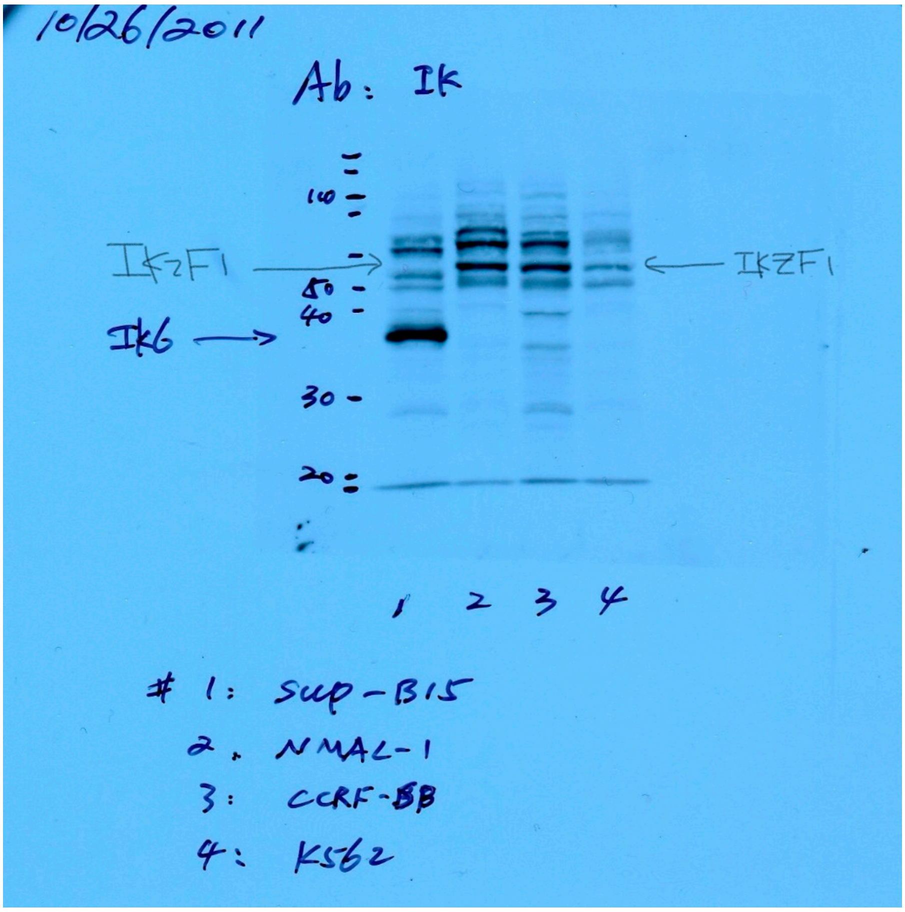

D

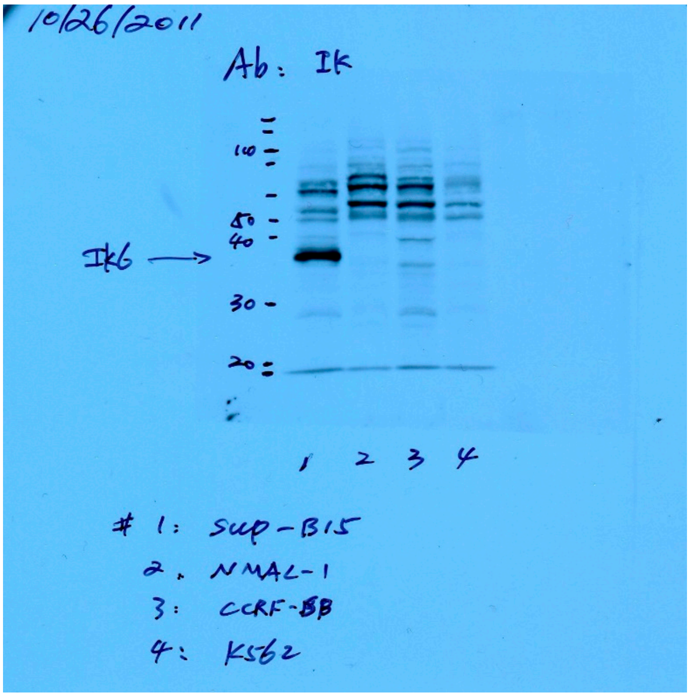

Figure S1. Uncropped Western Blotting figures of Figure 3.
